# Reference intervals of anti‐Müllerian hormone in Korean women

**DOI:** 10.1002/jcla.24525

**Published:** 2022-07-19

**Authors:** Rihwa Choi, Sang Gon Lee, Eun Hee Lee

**Affiliations:** ^1^ Department of Laboratory Medicine Green Cross Laboratories Yongin Korea; ^2^ Department of Laboratory Medicine and Genetics, Samsung Medical Center Sungkyunkwan University School of Medicine Seoul Korea; ^3^ Green Cross Laboratories Yongin Korea

**Keywords:** anti‐Müllerian hormone, health checkup, Korea, ovarian reserve, reference interval

## Abstract

**Background:**

Limited data are available with reference intervals of serum anti‐Müllerian hormone (AMH) level in Korean women.

**Methods:**

We retrospectively reviewed serum AMH test results performed with automated electrochemiluminescence immunoassay in Korean women who visited health promotion centers between January 2019 and December 2020. Serum AMH results by age group were compared with previously reported reference intervals.

**Results:**

During the 2‐year study period, a total of 1953 AMH test results from Korean women (age 20–49 years) undergoing general health checkups were obtained. Serum AMH level differed significantly by age group. Peak AMH level was observed at age 25–29 years and decreased to undetectable for subjects older than 44 years. The 2.5th, 5th, and 10th percentile values of the present study were comparable with previously assessed lower limits of reference intervals. The upper limit of the reference interval defined as the 97.5th percentile value in women younger than 35 years was higher than that of Western populations. The 90th percentile value of the present study population was similar to the 95th or 97.5th percentile value of reference intervals for Western populations of women younger than 35 years.

**Conclusion:**

Understanding patient populations and differences in reference intervals by age group and measurement method can help guide clinical decisions and clinical laboratory analysis.

## 1 INTRODUCTION

Anti‐Müllerian hormone (AMH) is originally described in the context of sexual differentiation in the male fetus, in which AMH is secreted as a 140 kDa dimeric glycoprotein hormone structurally related to transforming growth factor β and inhibin.^1^, ^2^ Anti‐Müllerian hormone in women is produced by granulosa cells of small, growing follicles in the ovary, in which serum AMH level strongly correlates with the number of growing follicles.^1^ Serum AMH in women has received increasing attention as an indicator for ovarian reserve. Its clinical use has increased in obstetrics and gynecology clinics for fertility treatment and to manage diseases of the reproductive system.^1^, ^2^

Serum AMH level is determined in clinical laboratories by manual assays using enzyme‐linked immunosorbent assays (ELISA) with Gen II (Beckman Coulter), Ultra‐sensitive AMH/MIS (Ansh Laboratories), or MenoCheck picoAMH (Ansh Laboratories) assay and by automated assays, such as Access AMH (Beckman Coulter), Elecsys AMH (Roche), and AFIAS AMH (Boditech Med).^1^, ^3^, ^4^ Because these assays detect different AMH isoforms using different antibody pairs, direct comparison of AMH values obtained by these assays remains problematic.^1^, ^3^ Global standardization of serum AMH measurement remains insufficient; different analytical platforms give rise to different AMH concentrations.^3^, ^4^, ^5^ Even though the World Health Organization (WHO) reference material for the international standard (IS) by the National Institute for Biological Standards and Control (NIBSC) exists, differences between analytical method results are observed.^1^, ^3^, ^6^

Previous studies concerning reference intervals of serum AMH assessed in Korean women used data from the Access (Beckman Coulter) or Gen II assay (Beckman Coulter), which had different calibration traceability from the Roche Elecsys assay, or data were obtained from limited numbers of subjects.^4^, ^7^, ^8^, ^9^, ^10^, ^11^

Therefore, we aimed to investigate serum AMH levels in Korean women of different age groups for use as reference intervals and to compare the results with reference intervals described in previous studies.

## 2 MATERIALS AND METHODS

We retrospectively reviewed data obtained through the laboratory information system of Green Cross Laboratories between January 2019 and December 2020 of Korean women aged 20–49 years who underwent serum AMH testing during health checkups performed in visiting health promotion centers. All data were anonymized prior to statistical analysis. Repetitive serum AMH results measured in the same individuals were excluded for statistical analysis. Serum AMH results were investigated by age group as follows: 20–24, 25–29, 30–34, 35–39, 40–44, and 45–49 years.

Serum AMH was analyzed using an automated electrochemiluminescence Elecsys AMH immunoassay (Roche, Mannheim, Germany) on an e411 analyzer (Roche). The analytical measurement range of the assay was 0.01–23.0 ng/ml. Accuracy of the AMH assay was verified by a proficiency testing/quality management program of a College of American Pathologists survey in the United States and by the Korean Association of External Quality Assessment Service.^6^, ^12^

The non‐parametric test was adopted when appropriate for non‐normally distributed continuous variables to compare serum AMH levels by age group. Serum AMH data were presented as 2.5th, 5th, 10th, median, 90th, 95th, and 97.5th percentile values.^13^ Statistical analysis was executed using MedCalc Statistical Software version 19.1.5 (MedCalc Software bv, Ostend, Belgium; https://www.medcalc.org; 2020). *p* values were considered significant at the 0.05 level.

Previous studies about serum AMH reference intervals in Korean women were reviewed in parallel with the results of the present study. Because clinical information of study subjects was limited in the present study, data from a public database for the entire Korean population by Statistics Korea (KOrean Statistical Information Service; KOSIS, https://kosis.kr/eng/) were collected. In addition, another public database for annual numbers of patients diagnosed with and managed for irregular menstrual cycle in Korea was reviewed through Healthcare Bigdata Hub by the Health Insurance Review & Assessment Service (HIRA) using the 10th revision, Clinical Modification of the International Statistical Classification of Diseases and Related Health Problems (ICD‐10‐CM) code N91 for absent, scanty, and rare menstruation and code N92 for excessive, frequent, and irregular menstruation (available at: http://opendata.hira.or.kr/op/opc/olap4thDsInfo.do).

This study was conducted according to the guidelines outlined in the Declaration of Helsinki, and all procedures involving human subjects were approved by the Institutional Review Board of Green Cross Laboratories (GCL‐2022‐1013‐01).

## 3 RESULTS

**FIGURE 1:**
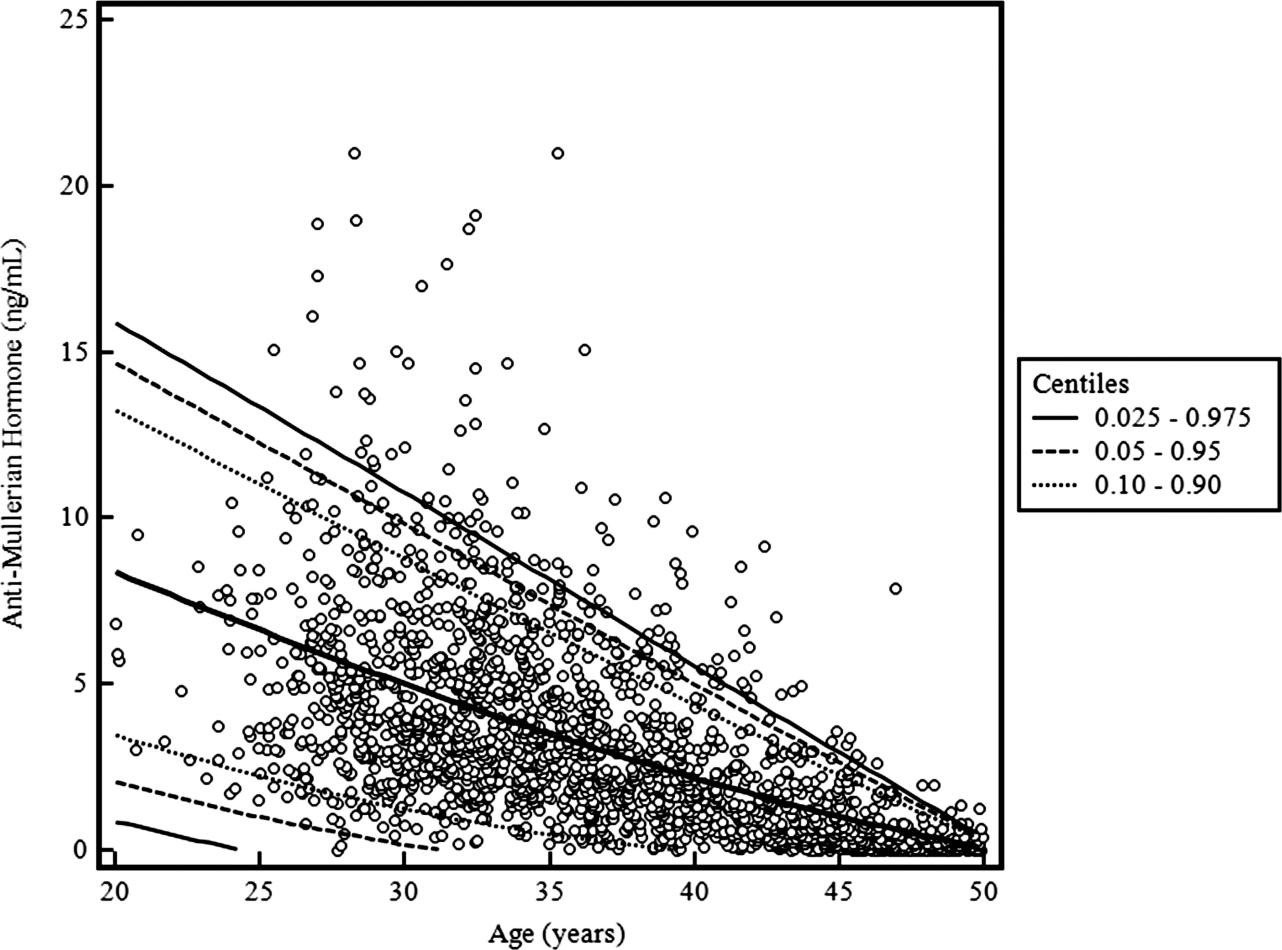
Serum AMH level by age in 1953 Korean women

During the 2‐year study period, a total of 1953 AMH test results from Korean women (age 20–49 years) undergoing general health checkups were obtained. Numbers of study subjects and AMH level by age group are summarized in Table 1 and Figure 1.

**TABLE 1:** Serum AMH level by age group in different studies

Age (years)	This study (Elecsys, Roche; Korean)								Manufacturer provided (Elecsys, Roche; Western population)						Anckaert et al. (Elecsys, Roche; Western population)^12^					
	*N*	2.5th	5th	10th	Median	90th	95th	97.5th	*N*	5th	10th	Median	90th	95th	*N*	2.5th	5th	Median	95th	97.5th
20–24	37	1.60	1.78	2.22	5.91	8.52	9.57	10.11	115	1.66	1.88	3.97	7.29	9.49	150	1.22	1.52	4.00	9.95	11.70
25–29	321	0.88	1.28	1.82	4.70	9.67	11.83	14.24	142	1.18	1.83	3.34	7.53	9.16	150	0.89	1.20	3.31	9.05	9.85
30–34	563	0.87	1.27	1.62	3.81	8.04	9.70	10.67	110	0.67	0.95	2.76	6.70	7.55	138	0.58	0.71	2.81	7.59	8.13
35–39	444	0.22	0.42	0.75	2.52	6.10	7.50	8.51	57	N/A	0.78	2.05	5.24	N/A	138	0.15	0.41	2.00	6.96	7.49
40–44	320	0.03	0.08	0.16	1.07	3.31	4.94	5.61	41	N/A	0.10	1.06	2.96	N/A	142	0.03	0.06	0.88	4.44	5.47
45–49	268	0.01	0.01	0.01	0.21	1.15	2.00	2.56	28	N/A	0.05	0.22	2.06	N/A	169	0.01	0.01	0.19	1.79	2.71

Age (years)	Kim et al. (Access, Beckman Coulter; Korean)^7^			Han et al. (Elecsys, Roche; Korean)^4^				Song et al. (Elecsys, Roche; Korean)^10^				
	*N*	2.5th	97.5th	*N*	10th	Median	90th	*N*	2.5th	Median	95th	97.5th
20–24	N/A	N/A	N/A	44	1.45	4.19	6.39	161	0.02	3.5	12.4	13.2
25–29	1486	0.75	13.38									
30–34	4007	0.41	10.64	112	1.25	3.44	6.29					
35–39	3900	0.12	8.18	98	0.61	1.97	5.47	126^a^	0.01	1.00	5.30	6.60
40–44	2220	N/A	4.91	39	0.10	1.36	4.17					
45–49	1190	N/A	1.93	N/A	N/A	N/A	N/A	N/A	N/A	N/A	N/A	N/A

Abbreviations: N, number; N/A, not available.

^a^ Only statistics for women 35–46 years old were available.

Among age groups, the number of tested subjects was highest in women aged 30–34 years. The number of women aged 20–24 years was <120. Serum AMH level was significantly different by age group (*p* < 0.0001). In post hoc analysis, the serum AMH levels in women aged 20–24 years and in women aged 25–29 years were not significantly different (*p* ≥ 0.05). The serum AMH levels in women from other age groups differed significantly from each other after post hoc analysis (*p* < 0.05).

The peak serum AMH level was observed at ages 25–29 years and declined to undetectable for subjects older than 44 years (45–50 years). In young age groups, the serum AMH level was widely distributed. The 2.5th, 5th, 10th, 90th, 95th, and 97.5th percentile values of serum AMH of the present study population were reviewed in parallel with previously described reference intervals and their definitions (Table 1).

**FIGURE 2:**
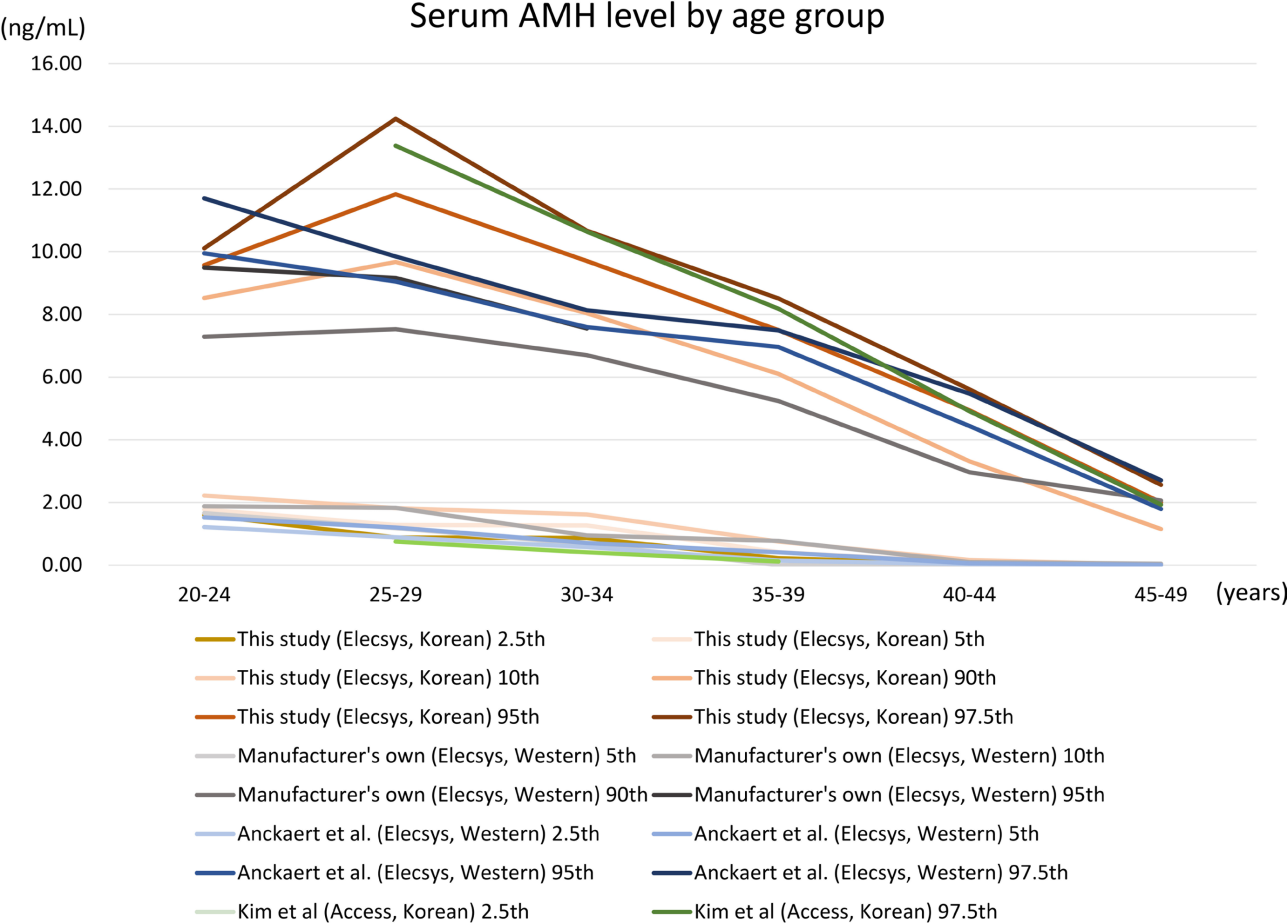
Serum AMH level by age group in different studies with more than 300 women (manufacturer's study, Anckaert et al.^12^ using the Elecsys assay, and Kim et al.^7^ using the Access assay)

Because studies performed using large numbers of subjects were limited, visualization for data comparison was applied for studies including more than 300 subjects and is presented in Figure 2. Overall, the 97.5th percentile value (which is the upper value for the central 95th percentile) of the present study was the highest of reported values with regard to the upper limit of reference intervals, except for women aged 45–49 years.

## 4 DISCUSSION

In this study, we investigated the distribution of serum AMH levels as determined using the automated Elecsys assay in different age groups of Korean women using data from adults who visited health promotion centers to receive health checkups. Previous studies performed with Korean women to determine reference intervals used data from analytical methods with different traceability or included only a limited number of subjects.^4^, ^7^, ^9^, ^10^ Considering the differences in absolute AMH values among analytical methods, results of the present study provide important information for use in clinical laboratories and for clinical decision making in fertility treatment.^1^, ^3^, ^6^

In this study, the number of women aged 20–24 years was less than 120, which was too few to apply the central 95th percentile value as a reference interval.^13^ Previous studies performed on Korean women did not include women aged 20–24 years or included limited numbers of study subjects of this age group, whose data were combined with those of women aged 25–29 years (Table 1).^4^, ^7^, ^10^ The peak serum AMH level observed in the 25–29‐year age group and the decrease to undetectable in the 45–50‐year age group were comparable with previous findings that the serum AMH level starts to decline from age 25 years and reaches an undetectable level at menopause.^1^

The 2.5th, 5th, and 10th percentile values of the present study were comparable with previously assessed lower limits of reference intervals.^7^, ^12^ The 97.5th percentile value in the Korean population was similar to the 97.5th percentile value of the present study population, which was higher than reference intervals in Western populations.^7^, ^10^, ^12^ The 90th percentile value of the present study population was similar to the 95th percentile value of reference intervals provided by the manufacturer and to the 97.5th percentile value of reference intervals performed in Western populations using the Elecsys assay by Anckaert et al.^12^ The upper limit of reference interval defined as the 97.5th percentile value in women younger than 35 years was higher than that of Western populations, while this limit was comparable among the other age groups (≥35 years). In this study, the presence of polycystic ovary syndrome (PCOS) or irregular menstrual cycle was not investigated. The presence of PCOS might affect the high 95th and 97.5th percentile values of the present study. According to HIRA and KOSIS, the prevalence of patients managed for an irregular menstrual cycle between 2019 and 2020 was about 9.0% of women aged 20–34 years and about 5.0% of women aged 30–49 years. Future studies including detailed clinical information of the reproductive system including PCOS or irregular menstrual cycle are needed.

The strength of this study is the large number of subjects in each age group used to investigate the serum AMH distribution using an Elecsys automated assay to assess the reference intervals. A limitation of this study is its retrospective design, which is vulnerable to preanalytical factors and the unknown homeostatic status of the subjects.^13^, ^14^ However, we investigated the possible prevalence of irregular menstrual cycle from public databases (HIRA and KOSIS). The results of our study might not be generalizable to other ethnic populations because all subjects in this study were Korean women. Future studies with a prospective design and detailed clinical information in association with ovarian reserve, menstrual cycle, and any disease associated with the reproductive system are needed to determine more accurate serum AMH level in a healthy population.^1^

In conclusion, we evaluated serum AMH level in Korean women visiting health promotion centers and compared them with previously reported reference intervals. The results of this study will be helpful to both physicians using serum AMH tests such as the Elecsys assay for patient management and to clinical laboratories. Future studies will focus on the reference interval and cutoff values of AMH including detailed clinical information in association with ovarian reserve.

## AUTHOR CONTRIBUTIONS

This work was performed as a collaboration among all authors. All authors accept responsibility for the entire content of this submitted article and approve its submission. All authors contributed to article preparation; R. Choi, S. G. Lee, and E. H. Lee collected the data or contributed to data analysis; R. Choi designed the study; R. Choi, S.G. Lee, and E. H. Lee had full access to all data in the study and take responsibility for the integrity of the data and the accuracy of the data analysis. All authors read and approved the final article.

## CONFLICT OF INTEREST

All authors declare no conflict of interest.

## PATIENT CONSENT STATEMENT

A waiver of informed consent was approved by the IRB (GCL‐2022‐1013‐01) since the study would not adversely affect the rights and welfare of the subjects due to its retrospective design and no more than minimal risk to the subjects.

## Data Availability

The data that support the findings of this study are available from the corresponding authors upon reasonable request.
